# Calisthenic Exercises for Girls

**Published:** 1888-12

**Authors:** 


### CALISTHENIC EXERCISES FOR GIRLS.

Calisthenic exercises, when conducted on scientific principles, are of great value in regulating the nerve centres; well co-ordinated series of movements imitated from the teacher, or produced to the word of command, tend to produce a well-knit nerve and muscular system responding with ease and gracefulness to impressions received through the eye and ear. The tendency to a symmetry of postures, and ill-balanced positions of the head and spine, with excess of movement, is often pronounced in growing girls, especially those of hysterical temperament; such conditions may be checked and brought under control through the eye and ear. Quickness of brain action and of the interaction of the senses and the hands, may be cultivated by exercises with balls. There is another advantage in such exercises: as the ball is thrown or caught by the child the eyes follow the moving object, as it recedes or advances towards her, and thus the power of accomodating the vision is brought into play. In young and delicate girls great care is necessary in using in such exercises as throw great strain upon special groups of muscles, and in the use of exercises designed to regulate brain action, fatigue should be carefully avoided, and throughout the lesson the signs of weakness and exhaustion should be carefully looked for, so that the strain may not be injuriously prolonged. To conduct such exercises and training with success, especially in weakly children, requires special training and care in the teacher.—*British Medical Journal.*

